# Experimental Study of Tensile Properties of Styrene–Butadiene–Styrene Modified Asphalt Binders

**DOI:** 10.3390/ma14071734

**Published:** 2021-04-01

**Authors:** Paweł Mieczkowski, Bartosz Budziński, Mieczysław Słowik, Jan Kempa, Wojciech Sorociak

**Affiliations:** 1 Faculty of Civil and Environmental Engineering, West Pomeranian University of Technology Szczecin, Al. Piastów 17, 70-310 Szczecin, Poland; Pawel.Mieczkowski@zut.edu.pl (P.M.); bbudzinski@zut.edu.pl (B.B.); 2 Faculty of Civil and Transport Engineering, Poznan University of Technology, Piotrowo 3, 60-965 Poznań, Poland; mieczyslaw.slowik@put.poznan.pl; 3 Faculty of Civil and Environmental Engineering and Architecture, UTP University of Science and Technology in Bydgoszcz, Al. Prof. S. Kaliskiego 7, 85-796 Bydgoszcz, Poland; janke@utp.edu.pl; 4 Faculty of Civil Engineering, Silesian University of Technology, Akademicka St. 2, 44-100 Gliwice, Poland

**Keywords:** asphalt binder, styrene–butadiene–styrene copolymer, force ductility test, rheology, modified bitumen

## Abstract

The requirements imposed on road pavements are ever increasing nowadays, necessitating the improvement of the properties of paving materials. The most commonly used paving materials include bituminous mixtures that are composed of aggregate grains bound by a bituminous binder. The properties of bitumens can be improved by modification with polymers. Among the copolymers used for modifying bitumens, styrene–butadiene–styrene, a thermoplastic elastomer, is the most commonly used. This article presents the results of tests conducted on bitumens modified with two types of styrene–butadiene–styrene copolymer (linear and radial). Two bitumen types of different penetration grades (35/50 and 160/220) were used in the experiments. The content of styrene–butadiene–styrene added to the bitumen varied between 1% and 6%. The results of the force ductility test showed that cohesion energy can be used for qualitative evaluation of the efficiency of modification of bitumen with styrene–butadiene–styrene copolymer. The determined values of the cohesion energy were subjected to the original analysis taking into account the three characteristic elongation zones of the tested binders. The performed analyses made it possible to find a parameter whose values correlate significantly with the content of styrene–butadiene–styrene copolymer in the modified bitumen. With smaller amounts of added modifier (approximately 2%), slightly better effects were obtained in the case of linear copolymer styrene–butadiene–styrene and for larger amounts of modifier (5–6%) radial copolymer styrene–butadiene–styrene was found to be more effective. This is confirmed by the changes in the binder structure, as indicated by the penetration index (PI).

## 1. Introduction

In any place in the world, bituminous mixtures—i.e., mixtures composed of aggregates and bituminous binders as the main components—are the most widely used road paving materials. The amount of binder relative to the total weight of the asphalt mixture is relatively small (from about 3.5% in asphalt concrete mixtures for road base courses up to as much as 7–8% in mastic asphalts). Despite the small amount, bitumen has a strong influence on the performance properties of pavements. The primary roles of the binder include creating stable joints between the grains of mineral aggregate [1,2], filling the empty spaces between them (air voids) and ensuring weather resistance of the mixture. In addition, bitumen or, to be more precise, its mixture with filler and fine sand (mastic) has some role in carrying the traffic loads.

With such a wide range of tasks to be performed by bituminous binders under continuously varying traffic conditions (including increase of loads) it becomes necessary to search for the ways to improve their performance properties. The desired improvements include, in the first place, reduction of temperature susceptibility, increase of viscosity and cohesion in the service temperature range and better bitumen/aggregate adhesion.

This can be obtained (at least to some extent) by means of bitumen modifiers. In Eastern and Central Europe, copolymers composed of an elastomer accompanied by a small amount of a plastomer are used in most cases due to the prevailing climatic conditions. Such copolymers must be verified for compatibility with the binder, i.e., must be capable of forming a uniform and stable physical mixture without affecting the colloidal stability of the bitumen [3,4]. Besides the compatibility of the constituents, an appropriate process must be used to obtain a good mixture. The factors that influence the properties of the modified bitumen include the modification time, i.e., the time for which the bitumen and polymer are stirred [5], the temperature of the process, and the stirring speed [6]. A change to the process can lead to chemical and physical changes to the bitumen and to the polymer, resulting in a loss of compatibility and stability of the final product [7,8].

One of the most popular copolymers used for modification of bituminous binders is thermoplastic elastomer styrene–butadiene–styrene (SBS) [9] developed in the 1960s [10]. The process of modification consists of three phases [11]: dispersion of polymer in a colloid mill, swelling of ultra-fine dispersion of polymer particles and maturing and, in the case of radial copolymer, cross linking, which takes place during cooling of the final product. In the swelling process the elastomer expands its volume several (up to nine) times [12], absorbing mainly the light fraction of bitumen [3,9,13,14].

The cross-linking process is reversible. This means that when the temperature rises above 120 °C the bonds between polybutadiene and polystyrene become broken which enables to process bitumen in hot mix asphalt, and afterwards the SBS network re-establishes during cooling. Strengthening of the SBS network becomes rapid after the modified bitumen has cooled down to below 100 °C [15,16]. This is due to the hardening of styrene domains, for which it is the glass transition temperature of styrene (T_g_) while for polybutadiene this temperature is −90 °C. These values of glass transition temperatures define the nature of the copolymer performance and behavior. When polymer modified bitumen is substantially overheated (to over 200–250 °C) both, polymer and bitumen completely and irreversibly decompose [17,18].

Modification of paving-grade bitumens with elastomers improves their elastic properties. The softening point increases, penetration decreases, viscosity increases in the service temperature range and the Fraass breaking point slightly decreases [19,20,21]. These changes considerably improve the properties of asphalt mixtures containing polymer modified bitumens [22], including higher resistance to permanent deformation [23,24], higher fatigue resistance [25,26,27] and better low temperature performance [28,29].

The properties of materials with rubber-like properties (including copolymers, such as SBS) can be represented by a two-dimensional stress–strain relationship based on the results obtained in the tension or compression tests, subject to maintaining a constant temperature of samples.

**Figure 1:**
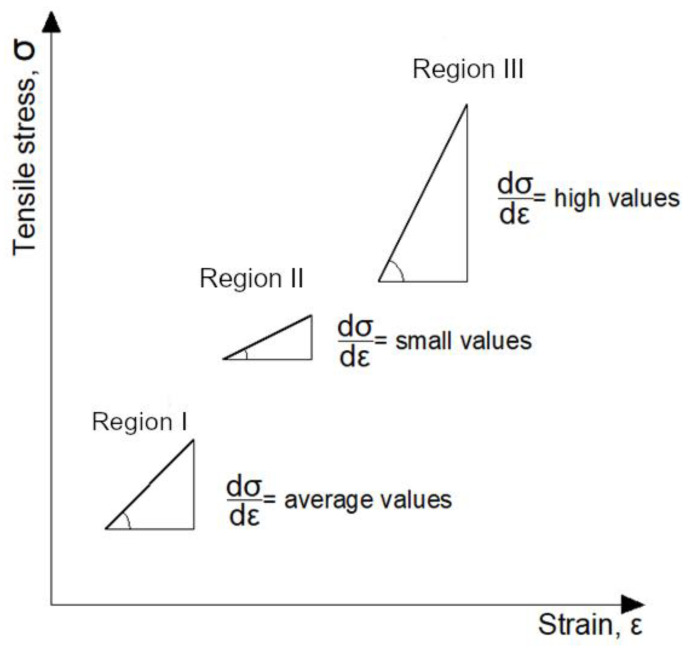
Stress–strain relationship during tensioning of materials featuring very high viscoelastic strain (elastomers). Adapted from ref. [30,31].

When a force is exerted on materials such as rubbers (both natural and synthetic), they behave differently than typical structural materials (steel, concrete, bitumen). This concerns both the mechanical properties and the energy states. Different behavior of these materials is closely related to their internal structure at the level of molecules, atoms and electrons. This behavior, accompanied by structural interpretation, is shown in Figure 1.

When analyzing the behavior of rubber-like materials attention should be paid to the following facts [4]:

1. The rheological flow curve represented by the stress–strain relationship exhibits three regions differing in terms of rheological (viscoelastic) behavior with different values of elasticity modulus assigned to them;
2. A mechanical property of the material, described by the material coefficient *E* (modulus of elasticity) reflects the changes taking place in the internal structure and depends on the deformation level:
  - Region I depicts the effect of exerting the force on a rubber sample whose hydrocarbon chains, to that moment randomly distributed throughout the volume, become oriented orderly, parallel to the force direction. The material offers a relatively high resistance to the force and the value of the modulus of elasticity is also relatively high (ca. 10 MPa);
  - Region II depicts the behavior of the material after the internal structure has been ordered—weak Van der Waals forces develop between parallel chains, which offer small resistance at this stage of deformation and the modulus of elasticity is very small (ca. 1 MPa);
  - Region III features very small strain increase levels in comparison to very high increases of stress due to covalent forces between atoms of carbon (C–C) in hydrocarbon chains, resulting in very high values of the modulus of elasticity (ca. 10 GPa);
3. After unloading the sample, the strain disappears rapidly, although also in the process of recovery three distinct steps can be identified, with immediate shortening of the sample in the first step and delayed subsequent steps.

The behavior of rubber-like materials and typical structural materials can also be analyzed on the basis of the thermodynamic function of state. Comparing steel and rubber in this respect, we find out that steel, being a crystalline material with regular structure, exhibits the lowest entropy before application of load. As the stress increases, in particular after the yield point, the entropy of this material starts to rise rapidly. It is the effect of the increase of internal energy and the degree of structural disorder of the body. This behavior of the system corresponds to irreversible processes. In the sample of rubber the disorder of hydrocarbon chains, i.e., the entropy of the material, is the greatest before the application of load and reaches the smallest level at the greatest stress value.

A rubber-like material in an unloaded state can be described as a chaotic tangle of very long and thin particles. At temperatures above the glass transition point polymer chains can undergo a so-called segmental motion. It is a coordinated change of the spatial arrangement (conformation) of groups composed of 3–5 meres (mere being the smallest repeating unit of a polymer). Owing to this segmental motion, the polymer chains can deform (e.g., straighten up) under the effect of an external force resulting in dimensional changes of the material they make up. This imposed *straightening up* of polymer chains decreases the entropy due to the increase of the structural order of the whole system. When the load is removed, the polymer chains start to twist spontaneously, also through segmental motion. The system tends to reach the highest possible entropy. Straightening of the polymer chains causes dimensional changes to the body of the material, and twisting returns it to the original shape.

**Figure 2:**
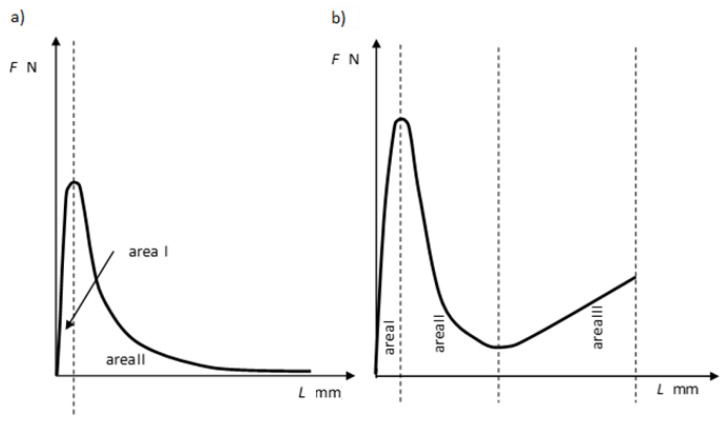
Example of relationship between the tensile force and elongation of a specimen of: (**a**) paving-grade (base) bitumen, (**b**) polymer-modified bitumen. Adapted from ref. [32].

Rubber-like materials are not used in road construction applications as a “pure” polymer. Instead, they are added as modifying additives. After mixing with the bitumen, they form various structures and phase systems depending on the quantity and the type of rubber and the consistency of bitumen with rheological properties halfway between bitumen (viscoelastic material) and rubber (material exhibiting mainly elastic properties). The changes to the rheological properties of the polymer-modified bitumen can be exemplified by cohesion determined from the relationship between the force (*F*) and elongation (*L*), obtained in the force ductility test. The effect of the polymer is clearly seen and manifested by changes to the relationship between the tensile force and the elongation of the binder specimen (Figure 2).

Area I is the elastic strain region of the analyzed bitumens. This area applies to relatively low strain values of the SBS modified binders (elongation of the binder specimen from 0 to 20 mm), and their behavior and the maximum tensile force obtained result from the properties (stiffness) of the base bitumen and the type and content of the SBS copolymer in the modified bitumen. In the case of polymer-modified binders, the surface area under the curve in this strain zone increases (the same as the maximum force value) with the increasing quantity of modifier in relation to the base bitumen. As the specimen elongation increases (Area II in Figure 2), the cross-sectional area decreases, reducing the force needed to further elongate the specimen. The observed decrease of the exerted force is smaller in the polymer-modified bitumens than in the base bitumens. This phenomenon can be explained by the superimposition of the effects of reducing the forces of resistance of the base bitumen and a systematic increase in the force of resistance of the crosslinked SBS copolymer. Further elongation (Area III in Figure 2b) increases the required force despite further reduction of the specimen cross-section. This reflects the stress–strain behavior of copolymer SBS. In the Area III, the influence of base bitumen properties on the tensile force values is negligible. Force ductility test is widely used method for bitumen testing. As presented in Figure 2, the behavior of both SBS modified and non-modified bitumen was analyzed by other researchers [33,34,35]. The results of ductility test (European version of double-edge-notched tension test) of the studies bear resemblance to the shape presented in Figure 2. The effect of bitumen modification is visible while elastomer is applied to bitumen. Tests revealed similar behavior for both crumb rubber (CR) and styrene–butadiene–styrene (SBS) modification, which is different from neat bitumen.

The above flow curves of “pure” elastomers (Figure 1) and bitumens modified with elastomer (Figure 2b) show that the elastomer contained in the bitumen increases the value of the modulus of elasticity, especially in Area II and Area III. This is caused mainly by the structural changes, which occur in the combined materials, namely in the bitumen and in the elastomer.

Determining the amount of copolymer used for asphalt binder modification is very difficult and requires specialized (very expensive) equipment. The test that indirectly assesses the effectiveness of polymer modification of bitumen is elastic recovery. However, it is difficult to estimate the amount of the modifier based only on the results of this study. The first studies with one type of SBS polymer and two types of bitumen (air-blown: 35/50 and 160/220, distilled: 35/50 and 160/220) were published in [36]. The obtained results show that the method can be effective in estimating the polymer content and is easy to perform. In the present paper, the determined cohesion energy values were subjected to the original analysis taking into account three characteristic zones of elongation of the tested binder specimens (Areas I, II and III in accordance with Figure 2b). The results of the analyzes made the authors look for a parameter whose values show a significant correlation with the content of the SBS copolymer in the modified asphalt binder.

## 2. Test Materials

Two base (air-blown) bitumens were chosen for the experiments: hard (35/50) and soft (160/220). Two varieties of SBS copolymer were used as the modifier: linear (SBS-L) and radial (SBS-R). 

The bitumens selected for the tests were produced at the domestic refinery (Poland) for the extraction of Ural Petroleum (Russia). Bitumen production was carried out by atmospheric and vacuum distillation of bitumen 160/220. The final stage of bitumen production was done by air-blowing of vacuum distillation residues in Biturox installations. The process relies on continuous supplying the reactors with raw material and constant receiving the final product. The oxidation reaction takes place in the entire volume of the binder in relatively short time and low air consumption.

For comparative purposes the bitumens used for the tests were heated in the conditions in which bitumen modification was carried out with SBS copolymers. Bitumen (in 10 dm^3^ capacity containers) was heated in an oil bath to 180 °C and kept at this temperature for 5 h.

**Figure 3:**
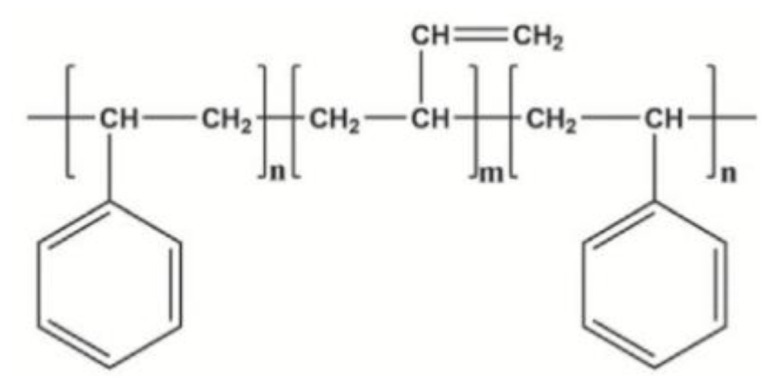
Structural formula SBS-L (high vinyl triblock). Reprinted from ref. [37].

**Figure 4:**
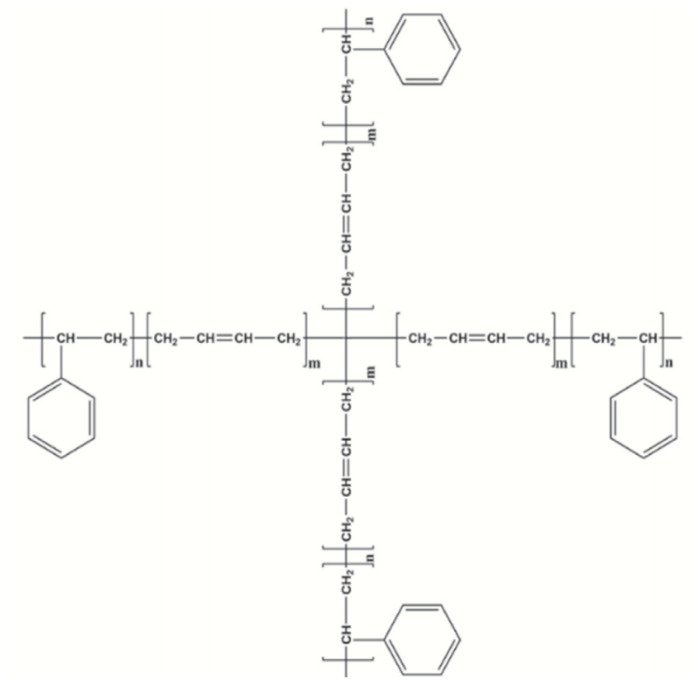
Structural formula SBS-R (radial triblock). Reprinted from ref. [37].

Styrene–butadiene–styrene (SBS) type copolymers have been widely used in the world since the 1970s. They are being used to improve the properties of the asphalt binder. As the part of the research the two types of Kraton company SBS copolymer were used: a linear block copolymer (D1192—marked as SBS-L in the tests) and a radial copolymer (D1184—marked as SBS-R). Properties of used copolymers are presented in Table 1 and the chemical (structural) formulas are presented in Figure 3 and Figure 4.

The differences in the composition of polymers can be assessed on the spectrometer basic tests FT-IR and NMR [37]. In the FT-IR, they can be observed mainly in terms of band 750–1200 cm^−1^. The increase in vinyl content significantly increases the intensity of the bands at 910 and 966 cm^−1^ due to =C–H vibrations in the polybutadiene (PB) segment [38,39]. The increased vinyl content can facilitate increased interaction between the polymer and the binder molecules due to the decreased steric hindrance to the C=C bond [37,38,39,40,41]. The assignment of the basic bands in the FT-IR spectrum is presented in Table 2.

The ^1^H NMR spectra of the polymers also show differences, with the signal at 5 ppm corresponding to the vinyl protons [37].

## 3. Modification of Bitumens with SBS Copolymers

Bitumens can be modified in order to obtain a binder with changed rheological properties, which can be desired, for example, to improve the performance of asphalt mixtures. This concerns both the low temperature performance (increased resistance to low-temperature cracking) and behavior at high service temperatures (improved resistance to permanent viscoplastic deformations).

**Figure 5:**
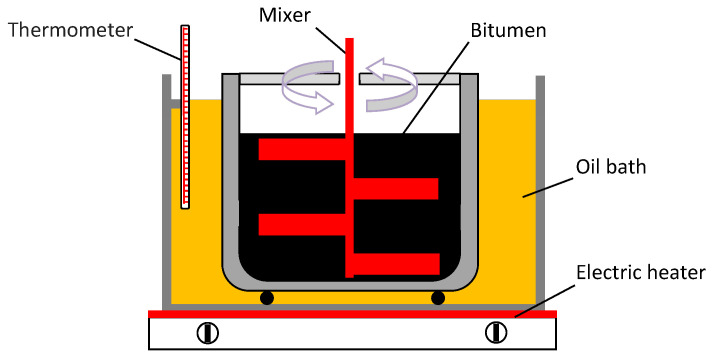
Modification of bituminous binder with SBS copolymer in oil bath.

The process of modification of the analyzed binders took place in a 10 dm^3^ metal container (sealed in order to limit access of air). The bitumen was heated up to 180 °C. This process was performed in an oil bath to ensure uniform warming of the bitumen container. After the specified temperature was reached, the modifier was added to the binder at the pre-determined proportion (from 1% to 6% weight of bitumen). Then the mix was stirred for 3 h at a constant speed of 120 rpm (the first 2–3 min at 1500 rpm) (Figure 5). The mixture of bitumen and copolymer was left to mature for 2 h at a constant temperature of 180 °C.

Before preparation of the test specimens the compatibility between the bitumen and the copolymer was checked. A so-called tube test was carried out to check phase separation of modified bitumen during storage at high temperatures. The determination was carried out according to EN 13399:2012 [42]. The tests were performed on bitumens 35/50 and 160/220 containing the highest applied amount (i.e., 6%) of SBS-L and SBS-R copolymers.

In the test, a uniform specimen of modified bitumen was kept for 72 h in a tube, in a vertical position, at a temperature of 180 °C. Next the specimen was cooled down and divided into three equal parts. The parameters determined for the two extreme parts (the top one and the bottom one) included penetration at 25 °C (according to EN 1426:2015-08) and T_R&B_ softening point (according to EN 1427:2015-08). The compatibility between the materials in question was carried out as per the requirements of PN-EN 14023:2011 [43] and the Polish guidelines TWT-PAD-2003 [44] (Table 3).

The results of the tube test are given in Table 4.

The differences between the values obtained for the bottom and upper parts of the samples for all the cases (Table 4) demonstrate stability of the bitumen-modifier system in consideration. Despite uncertainty in terms of penetration and softening point tests for different SBS type modification, both copolymers under analysis (SBS-L and SBS-R) show very good compatibility with the tested bituminous binders, both hard (35/50) and soft (160/220). In the case of the analyzed bitumens, slightly smaller differences were obtained for bitumen 35/50 and in the case of modifiers smaller differences were obtained for SBS-R copolymer.

The tube test results confirmed the suitability of the tested SBS copolymers for modification of the bitumens. The next twenty four samples of polymer-modified bitumen (containing 1% to 6% of each copolymer in increments of 1%) and two samples of base bitumens were prepared for further experiments.

## 4. Testing of Bituminous Binders

### 4.1. Penetration and Softening Point (T_R&B_) of the Tested Bitumens

The basic tests carried out in the case of bitumens included determination of the softening point T_R&B_ (according to EN 1427:2015-08 [45]) and penetration at 25 °C and 10 °C (according to EN 1426:2015-08 [46]). With the penetration values determined at these two temperatures it was possible to calculate the penetration index PI using Equations (1) and (2) [15].

(1) $$A=\frac{log\;P_{T_{1}}-logP_{T_{2}}}{T_{1}-T_{2}}$$

(2) $$PI=\frac{20-500A}{1-50A}$$

where:

$P_{T_{1}}$—penetration at temperature *T*_1_ (25 °C) [mm/10],

$P_{T_{2}}$—penetration at temperature *T*_2_ (10 °C) [mm/10].

### 4.2. Determination of Elastic Recovery

Elastic recovery is determined in the test (according to EN 13398 [47]) in which the sample of the tested bituminous binder is stretched in a ductilometer at a constant speed of 50 mm/min until the predetermined elongation length (200 mm) is obtained at a temperature of (25 ± 0.5) °C. In this way a bitumen thread is obtained, which is then cut in the middle to obtain two separate half-threads. After the pre-determined time (30 min.) the shortening of the half-threads is measured (i.e., the distance between the ends) and this value (*R_E_*) is expressed as a percentage of elongation, calculated using Equation (3).

(3) $$R_{E}=\frac{d}{L}\;\times \;100$$

where:

*R_E_*—elastic recovery (%),

*d*—distance between half-threads (mm),

*L*—elongation length of 200 mm.

### 4.3. Determination of the Tensile Force and Cohesion Energy in the Force Ductility Test

**Figure 6:**
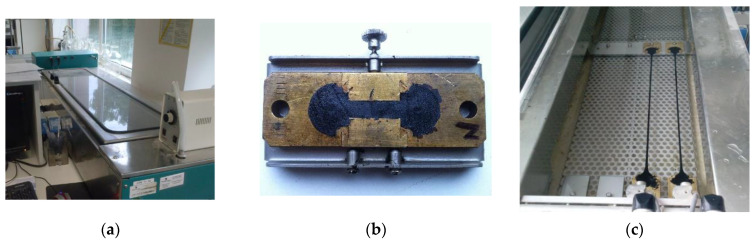
Force ductility test: (**a**) ductilometer, (**b**) test sample, (**c**) stretching of samples.

The tensile force and cohesion energy were determined according to EN 13589 [48] in a ductilometer (Figure 6). The apparatus was equipped with a tensile force measuring device with measurement range of 1 to 300 N (accuracy of ±0.1 N). The purpose of the test is to determine the extension capability of the material, taking account of the tensile force and the energy necessary to obtain a specific elongation value of the sample.

In the test a specially formed sample of material is stretched until rupture or 400 mm elongation (i.e., 1333% strain). The test was carried out at a constant speed of 50 ± 2.5 mm/min. The recommended test temperature is 5 °C and EN 14023 [34] prescribes three temperatures, depending on the type of bitumen tested, which are 5, 10 and 15 °C. Due to the problems with obtaining the required elongation and measurement repeatability, higher test temperatures were applied, namely 15 °C for bitumen 35/50 (base and modified) and 10 °C for bitumen 160/220 (base and modified).

According to EN 13589 [39] the cohesion energy ($E_{S}^{*}$) is calculated using equation (4) and it is the amount of energy used to obtain a 400 mm elongation, less the amount of energy used to obtain a 200 mm elongation.

(4) $$E_{S}^{*}=E_{b}^{*}-E_{a}^{*}$$

where:

$E_{S}^{*}$—value of the cohesion energy [J/cm^2^],

$E_{b}^{*}$—cohesion energy corresponding to the elongation of 0.400 m [J/cm^2^],

$E_{a}^{*}$—cohesion energy corresponding to the elongation of 0.200 m [J/cm^2^].

In addition, the amounts of energy corresponding to the respective phases were established from the surface areas under the force curve in the graph in Figure 2b (for polymer modified bitumens) [4].

- Area I ($E_{Area\;I}^{*}$),
- Area II ($E_{Area\;II}^{*}$),
- Area III ($E_{Area\;III}^{*}$).

Such energy analysis is not popular within the studies, as is it not a part of the standard EN 13589 [48]. The division of cohesion energy according to the stage of load application is an original approach to the bitumen test analysis. However, the division of the energy obtained from the double-edge-notched tension test was used in other studies for different purposes [49]. In the research, fracture energy was analyzed using ductility tests. The energy was divided into the energy necessary for the fracture process and the energy necessary for the plastic deformation outside.

## 5. Presentation and Analysis of the Results of Tests of Modified Bitumens

### 5.1. Penetration and Softening Point

The initial parameters of the bitumens used for the tests before heating at 180 °C (base) and after heating are presented in Table 5.

**Figure 7:**
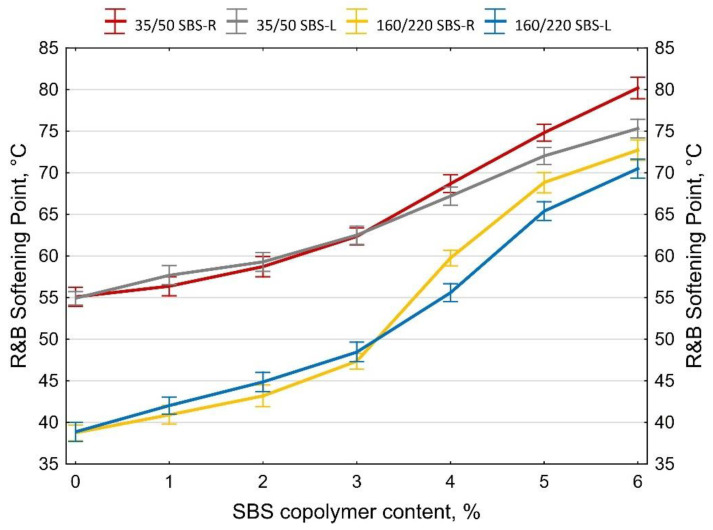
T**_R&B_** softening point of SBS-modified bitumens, error bars show a 95% confidence interval for the mean.

**Figure 8:**
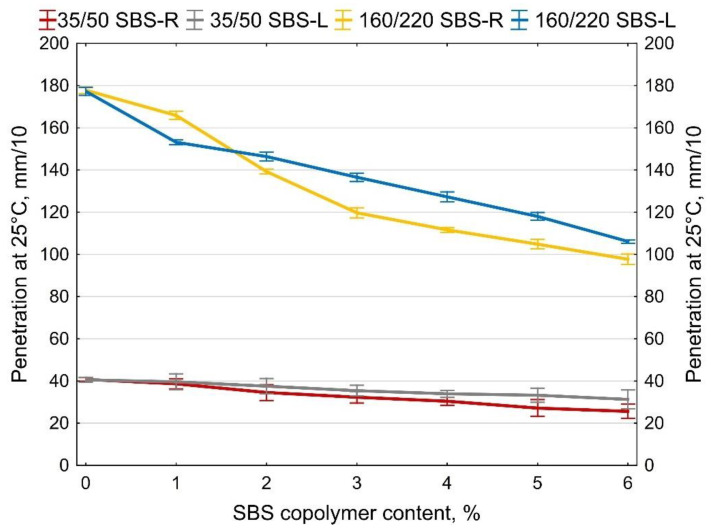
Penetration at 25 °C of SBS-modified bitumens, error bars show a 95% confidence interval for the mean.

**Figure 9:**
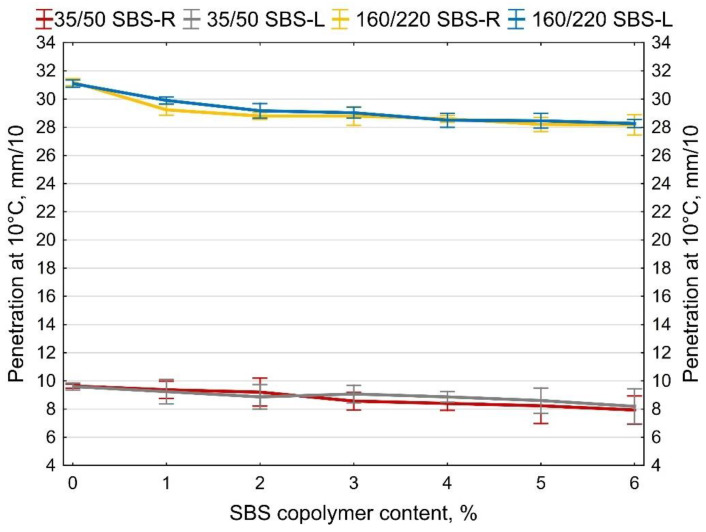
Penetration at 10 °C of SBS-modified bitumens, error bars show a 95% confidence interval for the mean.

**Figure 10:**
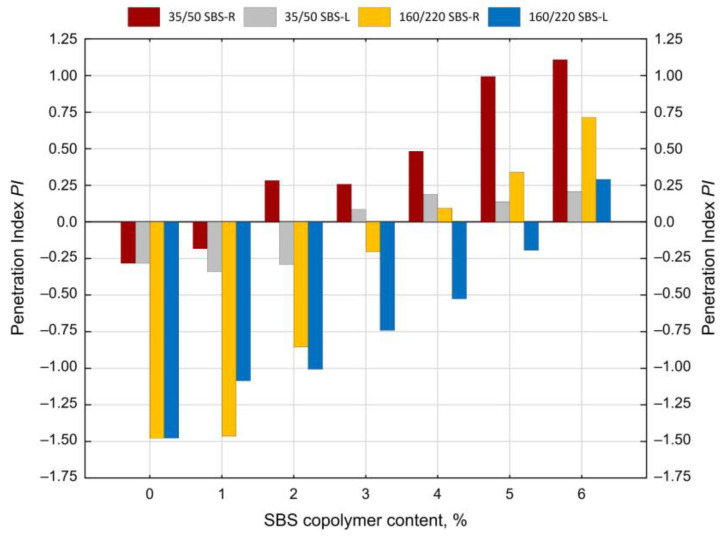
Penetration index of SBS-modified bitumens.

The determined softening point and penetration values at 10 and 25 °C are shown in Figure 7, Figure 8 and Figure 9. The values of the penetration index PI are given in Figure 10. Each value is an average of five measurement results.

The two types of SBS copolymer (radial and linear) improve the properties of the tested bitumens 35/50 and 160/220. The greatest changes (increases) were noted in the case of the softening point and penetration index values. The obtained results show that when the amounts of modifier are smaller (up to 2%) greater changes are obtained with SBS-L while SBS-R is more effective at greater amounts (ca. 5–6%).

In all the analyzed cases copolymer addition increased the penetration index from -1.5 (bitumen 160/220) and from −0.3 (bitumen 35/50) to +0.7 (bitumen 160/220 + 6% SBS-R) and to +1.2 (bitumen 35/50 + 6% SBS-R). The effects include significantly reduced temperature susceptibility of the tested binders.

### 5.2. Elastic Recovery

**Figure 11:**
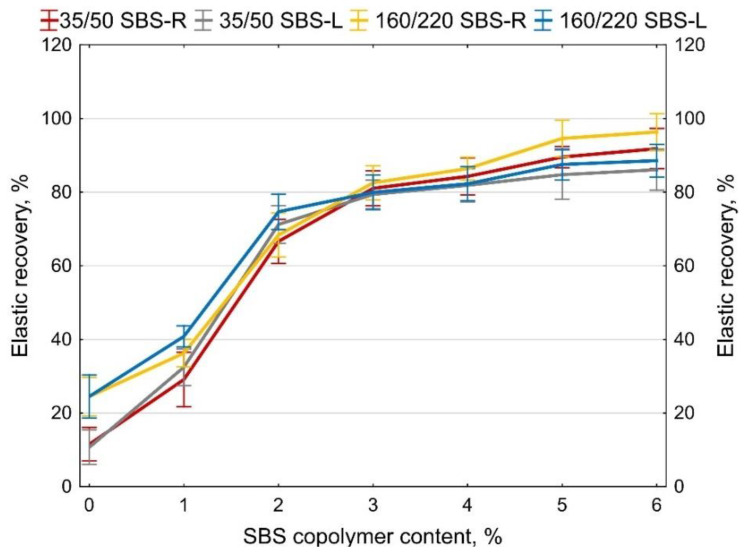
Elastic recovery of SBS-modified bitumens, error bars show a 95% confidence interval for the mean.

The values of elastic recovery obtained for the base and SBS-modified bitumens are given in Figure 11. Each presented value is an average of four measurement results.

According to [11], the elastic recovery of bitumens modified with a moderate amount of copolymer should not be lower than 50%. In the case of bitumens modified with high amounts of copolymer, this value should not be smaller than 70%.

Both copolymers (SBS-R and SBS-L) produce polymer-modified bitumens featuring good compatibility between the binder and the copolymer, satisfying the requirements of the applicable standards.

In the case of the tested polymer-modified bitumens, the elastic recovery requirements of 50% was considerably exceeded with as little as 2% content of SBS-L or SBS-R. At the amount of copolymer of 3%, elastic recovery of ca. 80% was recorded.

Paving grade base bitumens feature relative small values of elastic recovery, which is particularly true of the hard bitumen 35/50 (ca. 11%). Addition of up to 2% of copolymer considerably increased the elastic recovery, with the best effects obtained for SBS-L. A further increase of the modifier content causes a further increase of elastic recovery value, but the rate of change is smaller. At the content of polymer of 4% (and more) the cross-linking effect becomes apparent, obtained with copolymer SBS-R (binders obtain higher values of elastic recovery).

### 5.3. Tensile Force and Cohesion Energy

**Figure 12:**
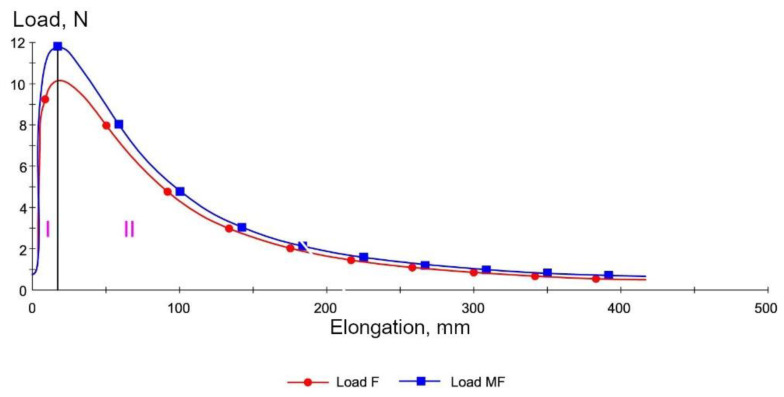
Load vs. elongation curves of paving grade bitumen 35/50.

**Figure 13:**
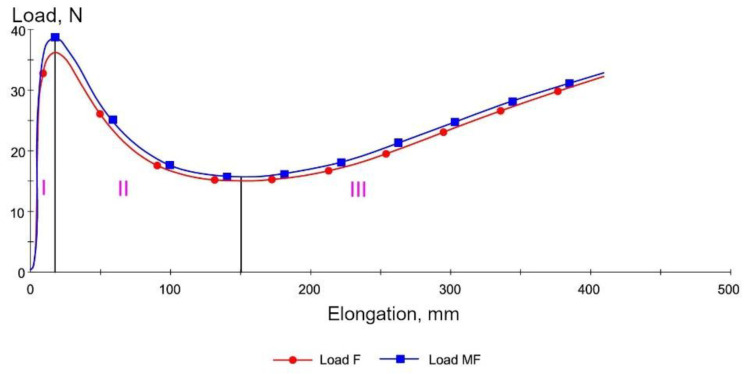
Load vs. elongation curves of bitumen 35/50 modified with 5% of SBS-R copolymer.

**Figure 14:**
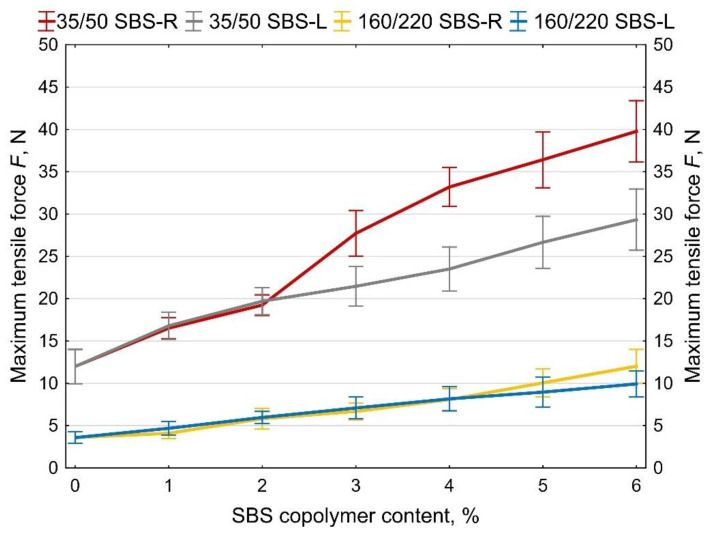
The maximum tensile force of SBS-modified asphalt binders determined using the force ductility test, error bars show a 95% confidence interval for the mean.

**Figure 15:**
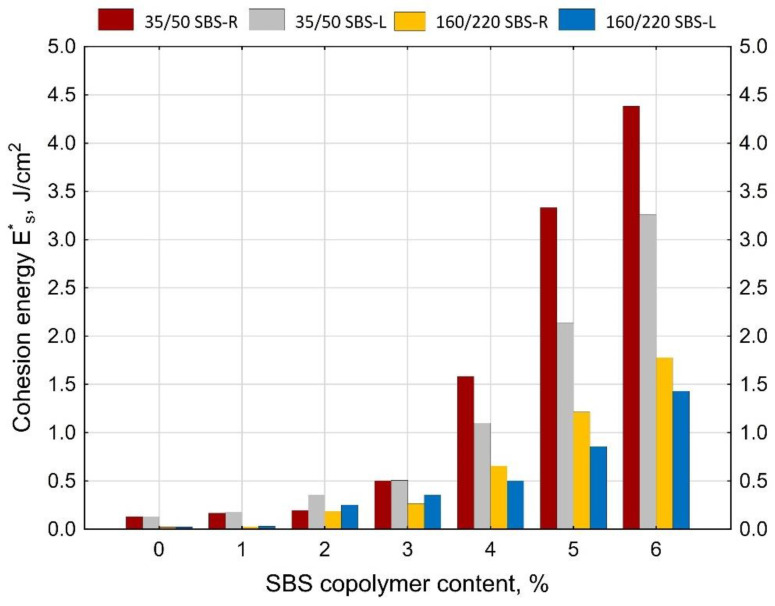
Cohesion energy ($E_{S}^{*}$) determined in the force ductility test.

**Figure 16:**
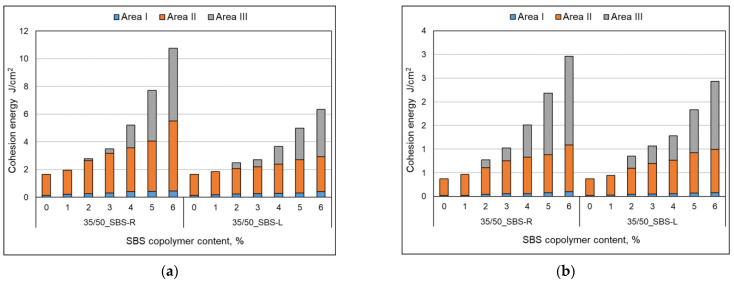
Cohesion energy of bitumens during stretching, taking account of the type of strain (as per Figure 2b), mean values, (**a**) bitumen 35/50, (**b**) bitumen 160/220.

**Figure 17:**
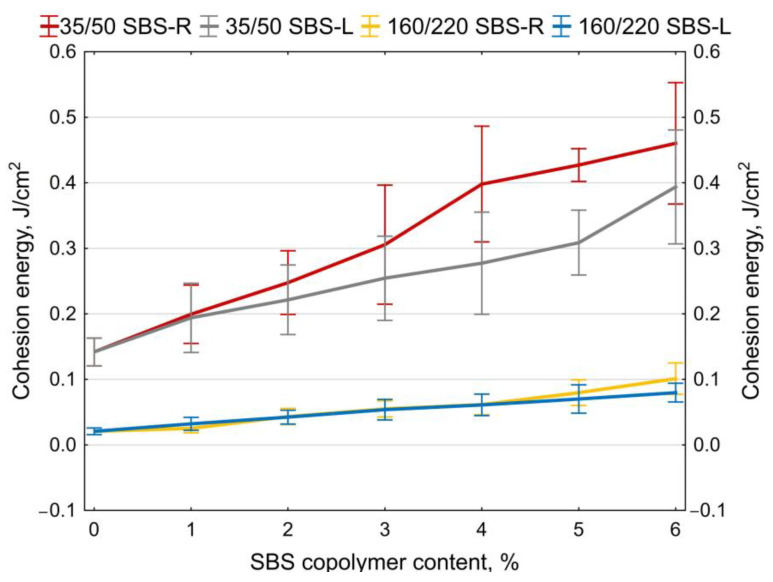
Cohesion energy of bitumen during stretching in Area I (as per Figure 2b), error bars show a 95% confidence interval for the mean.

**Figure 18:**
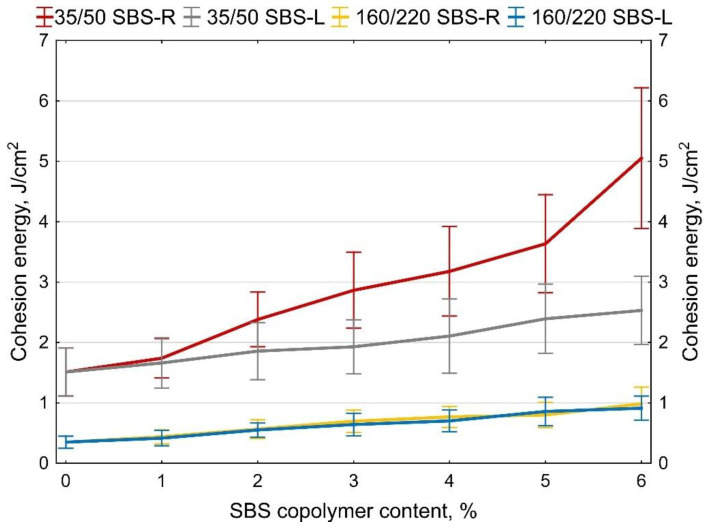
Cohesion energy of bitumen during stretching in Area II (as per Figure 2b), error bars show a 95% confidence interval for the mean.

**Figure 19:**
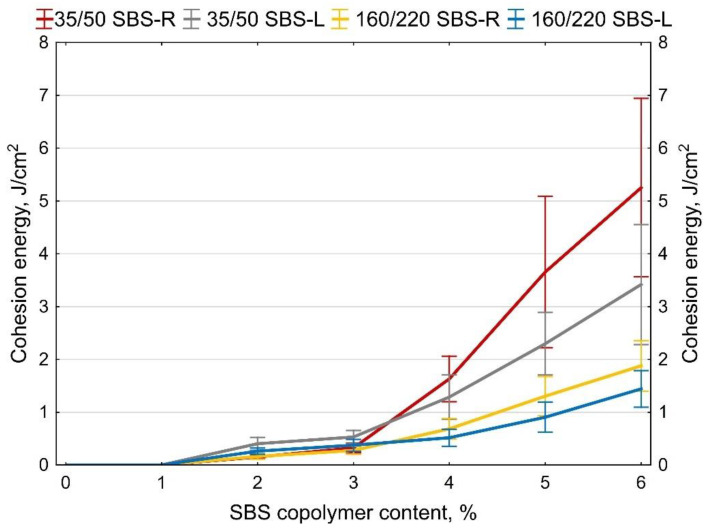
Cohesion energy of bitumen during stretching in Area III (as per Figure 2b), error bars show a 95% confidence interval for the mean.

Examples of measurement results obtained on two samples of 35/50 base bitumen and bitumen modified with 5% of SBS-R are shown in Figure 12 and Figure 13. The values of maximum tensile force F are compiled in Figure 14 and Figure 15. In addition, the cohesion energy values determined for the respective strain ranges (as defined in Figure 2b) are given in Figure 16, Figure 17, Figure 18 and Figure 19. Each presented value is an arithmetic mean of four measurement results.

Addition of SBS copolymer considerably increases the maximum tensile force value (Figure 14). With 6% content of copolymer the value of maximum force increases ca. 3 times in the case of radial copolymer (SBS-R) and between 2.2 (35/50) and 2.7 (160/220) times the initial values in the case of SBS-L. As to the cohesion energy (Figure 15) it is hard to judge the efficiency of modification, especially for the copolymer content of up to 3%. At greater amounts of copolymer the effect becomes apparent, especially in the case of hard bitumens.

Analyzing the cohesion energy values in the respective regions (as per Figure 2b) offers better evaluation of the efficiency of modification of bituminous binders with SBS copolymer. The greatest changes in the value of cohesion energy during stretching were noted in Area II (i.e., after reaching the maximum tensile force value) and in Area III (i.e., after reaching the minimum tensile force value). In Area I the changes are relatively small, falling in the range of 0.06 J/cm^2^ to 0.32 J/cm^2^ (at 6% content of modifier). In Area II the changes are bigger, especially in the case of bitumen 35/50 modified with SBS-R (the difference between the base bitumen and the bitumen modified with 6% of copolymer is 3.54 J/cm^2^, which is an increase by 234%). For the remaining cases these changes are in the range of 0.56 J/cm^2^ to 1.02 J/cm^2^ (increases in the range of 64% to 162% of the values obtained for the base bitumens).

The force ductility test results show that the behavior of base bitumens and bitumens containing small amount of modifier (up to 1%) does not include Area III (as per Figure 16, Figure 19), typical for rubber-like substances (Figure 2b). It is the region, which expands with the increase of the polymer amount. Small values of cohesion energy in Area III were noted at 2–3% content of SBS (irrespective of the SBS type). They fall in the range from 0.15 J/cm^2^ to 0.53 J/cm^2^. Much greater increases of the cohesion energy in Area III were obtained at copolymer amounts in excess of 4%. For 6% copolymer content these values fall in the range from 1.44 J/cm^2^ to 5.25 J/cm^2^. For both the tested bitumens (35/50 and 160/220) greater values of cohesion energy were obtained for modification with radial copolymer (SBS-R). This can be an indication of cross-linking of the polymer-modified bitumen.

**Figure 20:**
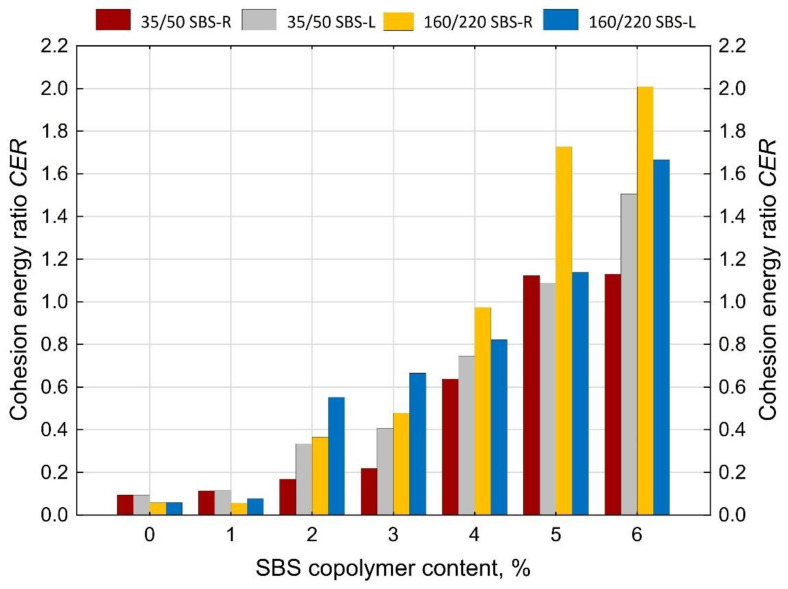
Values of the cohesion energy ratio (*CER*) done on the samples of polymer-modified bitumens in the force ductility test, calculated using Equation (5).

An interesting relationship was observed between the amount of SBS added to the bitumen and the cohesion energy ratio (*CER*) (Figure 20). The value of cohesion energy ratio (*CER*) was calculated using Equation (5).

(5) $$CER=\frac{E_{Area\;I}^{*}+E_{Area\;III}^{*}}{E_{Area\;II}^{*}}$$

where:

$E_{Area\;I}^{*}$ —cohesion energy in Area I as per Figure 2b [J/cm^2^],

$E_{Area\;II}^{*}$—cohesion energy in Area II as per Figure 2b [J/cm^2^],

$E_{Area\;III}^{*}$—cohesion energy in Area III as per Figure 2b [J/cm^2^].

With *CER* it is possible to determine the degree of modification of paving grade bitumens. The amount of modifier can be estimated on the basis of the value of cohesion energy ratio as follows:

- below 0.12—maximum 1% of SBS (or none at all),
- from 0.12 to 0.6—2–3% of SBS,
- from 0.6 to 0.9—ca. 4% of SBS,
- over 0.9—more than 5% of SBS.

**Figure 21:**
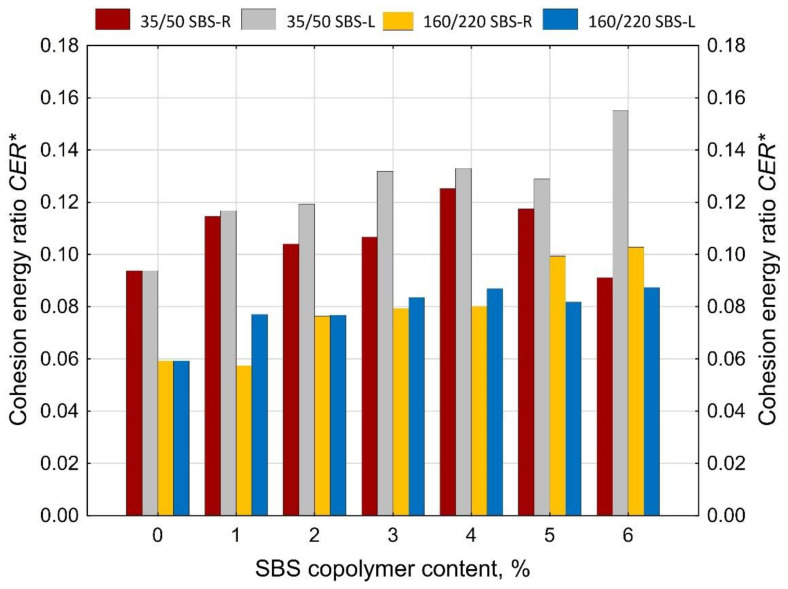
Values of CER* done on the samples of polymer-modified bitumens in the force ductility test, calculated using Equation (6).

For the sake of comparison Figure 21 gives the values of CER* described by Equation (6), which is the value of cohesion energy ratio done on the sample in the Area I and the cohesion energy done on the sample in the Area II. Irrespective of the amount of SBS the value of CER* does not exceed 0.16.

(6) $$CER^{*}=\frac{E_{Area\;I}^{*}}{E_{Area\;II}^{*}}$$

## 6. Conclusions

1. The basic parameters used to assess the quality of modification (besides the typical ones, i.e., the softening point and penetration) are elastic recovery and cohesion energy, which are determined by means of a ductilometer test. EN 14,023 estimates the cohesion on the basis of the difference between the cohesion energy determined at 400 mm extension ($E_{b}^{*}$) and cohesion energy at 200 mm extension ($E_{a}^{*}$). However, with this approach it is not possible to assess the nature of strain of the tested binder or to estimate the share of energy in Area III, characteristic of polymer-modified bitumens.
2. The force ductility test results show that more precise requirements regarding cohesion energy corresponding to the respective strain regions (Figure 2b) could be used as an additional criterion for assessing the efficiency of modification. Particular attention should be paid to the ratio between the sum of cohesion energy determined in the Area I and Area III and the cohesion energy determined in Area II (Figure 20), which can provide an indirect indication of the modifier content. This is particularly important for the pavement behavior at high temperatures in summer. A transition of the strain type of the binder (and also the asphalt mixtures containing it) to viscoelastic at higher levels of strain will be a factor improving the performance parameters of the pavement, including in particular resistance to permanent viscoplastic deformation.
3. The test results show that first qualitative changes to the behavior of binders can be observed with only 2% of modifier added, and this concerns both hard (35/50) and soft (160/220) bitumens and the two types of SBS copolymer, namely SBS-L (linear structure) and SBS-R (radial structure). This is particularly evident in Figure 19 and Figure 20. The decisive factor is the appearance of viscoelastic strains in the stretched sample (Area III), which is caused by the presence of SBS copolymer.
4. Determining the amount of polymer used for asphalt binder modification is very difficult and requires specialized (very expensive) equipment. The proposed original method can estimate the polymer content in the asphalt binder in a simple but effective way.

## Figures and Tables

**Table 1:** Properties of linear copolymer styrene–butadiene–styrene (SBS)-linear (L) (D1192) and radial copolymer SBS-radial (R) (D1184), (Kraton Company Data Document).

Property	Test Method	SBS-L	SBS-R
Specific Gravity	ISO 2781	0.94	0.94
Bulk Density, kg/dm^3^	ASTM D 1895 method B	0.4	0.4
Hardness (15 s/30 s), Shore A	ASTM D 2240	70 ^1^	75 ^2^
Elongation at Break, %	ISO 37	1000	820
Melt Flow Rate (200 °C/5 kg), g/10 min	ISO 1133	<1	<1
Tensile strength, MPa	ISO 37	33	27
300% Modulus, MPa	ISO 37	4.8	2.5
Polystyrene Content, %	KM 03	28.5–32.5	29.0–31.0
Vinyl Content, %	KM 03	≥35	≤15
Styrene/Butadiene Weight Ratio	KM 03	30/70	30/70
Molecular Weight kg/mol	KM 01	138–162	–
Viscosity in Toluene at 25 °C:25 wt %, Pa∙s	KM 06	1.4–2.4	1.0–1.4
Triblock content, %	KM01	≥90	–
Antioxidant content, %	KM08	≥0.16	≥0.14

^1^ Hardness—15 s; ^2^ hardness—30 s.

**Table 2:** Assignment of the main bands in FT-IR spectra of the SBS polymers Adapted from ref. [37].

Wavenumber (cm^−1^)	Compound	Origin (α—Bending, β—Stretching)
699, 750	PS	C–H (α-Aromatic ring)
650–750	PB	=C–H (α-Cis 1,4)
910	PB	=C–H (α-Cis 1,2)
966	PB	=C–H (α-Trans 1,4)
1450	PB	C–H (α-aliphatic –[CH_2_]_n_–])
1450–1500	PS	C–C (β-Aromatic ring)

**Table 3:** Permissible deviations for penetration and softening point (T_R&B_) values of polymer-modified bitumen determined in the tube test.

Type of Test	Maximum Absolute Value of the Difference According to	
	TWT-PAD-2003	PN-EN 14023:2011/Ap2:2020-02
Softening point (T_R&B_) (5 °C/min) (°C)	2	5
Penetration (25 °C, 100 g, 5 s), (mm/10)	5	NR

NR—no requirement.

**Table 4:** Penetration and softening point (T_R&B_) of polymer-modified bitumen obtained in the tube test.

Type of Modified Bitumen	Type of SBS	Type of Test					
		Penetration (mm/10)			Softening Point (T_R&B_) (°C)		
		Top Part of Sample	Bottom Part of Sample	Difference	Top Part of Sample	Bottom Part of Sample	Difference
35/50 +6%SBS	linear	30.5	28.4	2.1	75.8	77.3	1.5
	radial	24.9	23.3	1.6	80.6	81.6	1.0
160/220 +6%SBS	linear	108.7	105.2	3.5	71.4	73.2	1.8
	radial	100.4	98.1	2.3	73.2	74.4	1.2

**Table 5:** Parameters of the bitumens used for the tests (base and after heating 180 °C).

Type of Test	Standard	Bitumen			
		Base		After Heating	
		35/50	160/220	35/50	160/220
Penetration (25 °C, 100 g, 5 s.), (mm/10)	EN 1426:2015-08	45.4	188.7	40.6	177.3
Penetration (10 °C, 100 g, 5 s.), (mm/10)		9.8	31.5	9.6	31.1
Softening point (T_R&B_) (5 °C/min), (°C)	EN 1427:2015-08	54.5	37.1	54.7	38.6
Penetration index (PI), (−)	EN 12591:2010 (Annex A)	–0.36	–1.65	–0.28	–1.48

## Data Availability

Data is contained within the article.

## References

[1] Sengoz B., Isikyakar G. (2008). Evaluation of the Properties and Microstructure of SBS and EVA Polymer Modified Bitumen. Constr. Build. Mater..

[2] Kamiya S., Tasaka S., Zhang X., Dong D., Inagaki N. (2001). Compatibilizer Role of Styrene-Butadiene-Styrene Triblock Copolymer in Asphalt. Polym. J..

[3] Stefańczyk B., Mieczkowski P. (2009). Mieszanki mineralno-asfaltowe: Wykonawstwo i badania.

[4] Fu H., Xie L., Dou D., Li L., Yu M., Yao S. (2007). Storage Stability and Compatibility of Asphalt Binder Modified by SBS Graft Copolymer. Constr. Build. Mater..

[5] Larsen D.O., Alessandrini J.L., Bosch A., Cortizo M.S. (2009). Micro-Structural and Rheological Characteristics of SBS-Asphalt Blends during Their Manufacturing. Constr. Build. Mater..

[6] García-Morales M., Partal P., Navarro F.J., Martínez-Boza F.J., Gallegos C. (2007). Processing, Rheology, and Storage Stability of Recycled EVA/LDPE Modified Bitumen. Polym. Eng. Sci..

[7] Cortizo M.S., Larsen D.O., Bianchetto H., Alessandrini J.L. (2004). Effect of the Thermal Degradation of SBS Copolymers during the Ageing of Modified Asphalts. Polym. Degrad. Stab..

[8] Pérez-Lepe A., Martínez-Boza F.J., Gallegos C., González O., Muñoz M.E., Santamaría A. (2003). Influence of the Processing Conditions on the Rheological Behaviour of Polymer-Modified Bitumen☆. Fuel.

[9] Zhu J., Birgisson B., Kringos N. (2014). Polymer Modification of Bitumen: Advances and Challenges. Eur. Polym. J..

[10] Wypych G. (2012). Handbook of Polymers.

[11] Błażejowski K., Styk S. (2004). Technologia Warstw Asfaltowych.

[12] Valkering C.P., Vonk W.C., Whiteoak C.D. (1992). Improved Asphalt Properties Using SBS Modified Bitumen. Rev. Gen. Des. Routes Et Des. Aerodr..

[13] Porto M., Caputo P., Loise V., Eskandarsefat S., Teltayev B., Oliviero Rossi C. (2019). Bitumen and Bitumen Modification: A Review on Latest Advances. Appl. Sci..

[14] Airey G. (2003). Rheological Properties of Styrene Butadiene Styrene Polymer Modified Road Bitumens⋆. Fuel.

[15] Read J., Whiteoak D., Hunter R.N. (2003). The Shell Bitumen Handbook.

[16] Isacsson U., Lu X. (1995). Testing and Appraisal of Polymer Modified Road Bitumens—State of the Art. Mater. Struct..

[17] Singh S.K., Kumar Y., Ravindranath S.S. (2018). Thermal Degradation of SBS in Bitumen during Storage: Influence of Temperature, SBS Concentration, Polymer Type and Base Bitumen. Polym. Degrad. Stab..

[18] Sarnowski M., Kowalski K., Król J., Radziszewski P. (2019). Influence of Overheating Phenomenon on Bitumen and Asphalt Mixture Properties. Materials.

[19] Kumar P. (2012). Evaluation of Physical Properties of SBS Modified Bitumen and Effect of Aging. Chemical Engineering.

[20] Zhuang C., Li N., Zhao W., Cai C. (2017). Effects of SBS Content on the Performance of Modified Asphalt. Iop Conf. Ser. Mater. Sci. Eng..

[21] Zhang C., Wang H., You Z., Gao J., Irfan M. (2019). Performance Test on Styrene-Butadiene-Styrene (SBS) Modified Asphalt Based on the Different Evaluation Methods. Appl. Sci..

[22] Nikolaidēs A. (2017). Highway Engineering: Pavements, Materials and Control of Quality.

[23] Sirin O., Kim H.-J., Tia M., Choubane B. (2008). Comparison of Rutting Resistance of Unmodified and SBS-Modified Superpave Mixtures by Accelerated Pavement Testing. Constr. Build. Mater..

[24] Tayfur S., Ozen H., Aksoy A. (2007). Investigation of Rutting Performance of Asphalt Mixtures Containing Polymer Modifiers. Constr. Build. Mater..

[25] Li K., Huang M., Zhong H., Li B. (2019). Comprehensive Evaluation of Fatigue Performance of Modified Asphalt Mixtures in Different Fatigue Tests. Appl. Sci..

[26] Kim T.W., Baek J., Lee H.J., Choi J.Y. (2013). Fatigue Performance Evaluation of SBS Modified Mastic Asphalt Mixtures. Constr. Build. Mater..

[27] Kluttz R., Willis J.R., Molenaar A., Scarpas T., Scholten E., Scarpas A., Kringos N., Al-Qadi I.A.L. (2012). Fatigue Performance of Highly Modified Asphalt Mixtures in Laboratory and Field Environment. Proceedings of the 7th RILEM International Conference on Cracking in Pavements.

[28] Isacsson U., Zeng H. (1998). Low-Temperature Cracking of Polymer-Modified Asphalt. Mater. Struct..

[29] Lin P., Huang W., Li Y., Tang N., Xiao F. (2017). Investigation of Influence Factors on Low Temperature Properties of SBS Modified Asphalt. Constr. Build. Mater..

[30] Gul W.E., Kuljezniew W.N. (1979). Struktura I Mjechaniczeskoje Swojstwa Polimierow.

[31] Mieczkowski P. (2006). Substancje Kauczukopodobne w MMA. Mag. Autostrady.

[32] Gaweł I., Kalabińska M., Piłat J. (2014). Wydawnictwa Komunikacji I Łączności Asfalty Drogowe.

[33] Zhang B., Chen H., Zhang H., Wu Y., Kuang D., Guo F. (2020). Laboratory Investigation of Aging Resistance for Rubberized Bitumen Modified by Using Microwave Activation Crumb Rubber and Different Modifiers. Materials.

[34] Paliukaite M., Assuras M., Hesp S.A.M. (2016). Effect of Recycled Engine Oil Bottoms on the Ductile Failure Properties of Straight and Polymer-Modified Asphalt Cements. Constr. Build. Mater..

[35] Xu J., Yang E., Luo H., Ding H. (2020). Effects of Warm Mix Additives on the Thermal Stress and Ductile Resistance of Asphalt Binders. Constr. Build. Mater..

[36] Mieczkowski P., Budzinski B. (2019). Evaluation of The Modification Efficiency of Bituminous Binders with SBS Polymer Based on Changes in Strain Energy in the Ductility Test. IOP Conf. Ser. Mater. Sci. Eng..

[37] Kumar Y., Singh S.K., Oberoi D., Kumar P., Mohanty P., Ravindranath S.S. (2020). Effect of Molecular Structure and Concentration of Styrene-Butadiene Polymer on Upper Service Temperature Rheological Properties of Modified Binders. Constr. Build. Mater..

[38] Canto L.B., Mantovani G.L., deAzevedo E.R., Bonagamba T.J., Hage E., Pessan L.A. (2006). Molecular Characterization of Styrene-Butadiene-Styrene Block Copolymers (SBS) by GPC, NMR, and FTIR. Polym. Bull..

[39] Masson J.-F., Pelletier L., Collins P. (2001). Rapid FTIR Method for Quantification of Styrene-Butadiene Type Copolymers in Bitumen. J. Appl. Polym. Sci..

[40] Wang K., Yuan Y., Han S., Yang Y. (2019). Application of FTIR Spectroscopy with Solvent-Cast Film and PLS Regression for the Quantification of SBS Content in Modified Asphalt. Int. J. Pavement Eng..

[41] Yan C., Huang W., Xiao F., Wang L., Li Y. (2018). Proposing a New Infrared Index Quantifying the Aging Extent of SBS-Modified Asphalt. Road Mater. Pavement Des..

[42] (2012). PN-EN 13399:2012 Bitumen and Bituminous Binders. Determination of Storage Stability.

[43] PN-EN 14023:2011/Ap2:2020-02 (2020). Bitumen and Bituminous Binders-Specification Framework for Polymer Modified Bitumens.

[44] Sybilski D. (2003). Tymczasowe Wytyczne Techniczne. Polimeroasfalty Drogowe (TWT-PAD-2003).

[45] EN 1427:2015-08 (2015). Bitumen and Bituminous Binders-Determination of the Softening Point-Ring and Ball Method.

[46] PN-EN 1426:2015-08 (2015). Bitumen and Bituminous Binders-Determination of Needle Penetration.

[47] PN-EN 13398:2017-12 (2017). Bitumen and Bituminous Binders-Determination of the Elastic Recovery of Modified Bitumen.

[48] PN-EN 13589:2011 (2011). Bitumen and Bituminous Binders-Determination of the Tensile Properties of Modified Bitumen by the Force Ductility Method.

[49] Andriescu A., Hesp S., Youtcheff J. (2004). Essential and Plastic Works of Ductile Fracture in Asphalt Binders. Transp. Res. Rec..

